# Porphin-Based Carbon Dots for “Turn Off–On” Phosphate Sensing and Cell Imaging

**DOI:** 10.3390/nano10020326

**Published:** 2020-02-14

**Authors:** Jing Wu, Wenjing Wang, Zonghua Wang

**Affiliations:** College of Chemistry and Chemical Engineering, Shandong Sino-Japanese Center for Collaborative Research of Carbon Nanomaterials, Qingdao University, Qingdao 266071, China; 18463758410@163.com (J.W.); wangwenjing@qdu.edu.cn (W.W.)

**Keywords:** carbon dots, porphin, cell imaging, phosphate detection

## Abstract

Porphin-based carbon dots (denoted as PCDs) are prepared through a one-step hydrothermal method by using meso-tetra (4-carboxyphenyl) porphin (TCPP), citric acid, and ethanediamine as precursor. PCDs give rise to the optimal photoluminescence at λ_ex_/λ_em_ = 375/645 nm, exhibit an excitation-independent property, excellent water solubility, and good biocompatibility, which provide red emission and avoid the autofluorescence as an efficient fluorescent imaging probe. On the other hand, when Eu^3+^ is added into PCDs, the carboxylate groups located on the surface of PCDs exhibit high affinity to Eu^3+^, resulting in the fluorescence of PCDs turning off via static quenching. In the presence of phosphate, owing to the strong coordination with Eu^3+^, the fluorescence of PCDs turns on. Based on this performance, a novel “turn off–on” phosphate sensing system is developed. The detection limit of this sensing system can attain 3.59 × 10^−3^ μmol L^−1^. This system has been utilized for the detection of phosphate in real samples successfully, which further demonstrates potential applications in biological diagnostic and environmental analysis.

## 1. Introduction

Carbon dots (CDs), as an emerging photoluminescent nanomaterial, have attracted widespread attention since their initial discovery in 2004 [1]. Due to their superior optical properties, outstanding biocompatibility, excellent dispersibility, facile surface functionalization, simple, and low-cost synthesis process [2], CDs have been demonstrated to be a future perspective fluorescence nanomaterial in various applications including bioimaging [3,4,5,6], drug delivery [7], chemical sensing [8,9,10,11,12], photocatalysis [13], and anti-counterfeiting [14].

CDs mostly show blue or green emission under the excitation of ultraviolet/blue light [15,16], which is not suitable for biological imaging/biomedicine because of high background signal, poor cell/tissue penetration, and damage to cell/tissue. This factor also restricts their development in optoelectronic devices, photocatalysis, and sensing. Thus, preparing long-wavelength/red-emissive CDs is of vital importance. According to the photoluminescent mechanism of CDs, red-emissive CDs can be obtained through regulating particle size [17,18], shifting the excitation wavelength on the basic of excitation-dependent emission [19,20], extending the size of isolated sp^2^ domains [21,22,23,24], enhancing surface oxidation degree [25,26], or adopting heteroatomic doping [27,28,29,30,31]. Among these, expanding conjugated aromatic π system has been approved to be an effective way to enhance the size of isolated sp^2^ domains [32]. Various kinds of precursors with aromatic structure, such as p-phenylenediamine [25], 1,3-dihydroxynaphthalene [33], 2,5-diaminotoluene sulfate [34], polythiophene derivatives [35], trinitropyrene [36], or other IR/NIR dye molecules [37], are employed to construct red-emissive carbon dots.

In addition, to tune the property of CDs, kinds of functionalization strategies are developed to satisfy the specific requirement and broaden their application. For example, photosensitizer chlorin e6 modified CDs for multimodal imaging and cancer photothermal/photodynamic therapy [38]; fluorescein conjugated CDs for ratiometric pH sensing [39]; transferrin conjugated CDs for drug delivery [40]; nitrogen-doped MoS_2_ and nitrogen-doped carbon dots composite developed as electrocatalysts for electroreduction [41]. Therefore, developing effective and controllable route to synthesize red-emissive CDs or CDs hybrid/complex/composite with other nanomaterials is still desirable and challenging in order to broaden the application fields mainly in photothermal therapy, fluorescence sensing, optoelectronic devices, bioimaging, and photocatalysis.

**Scheme 1:**
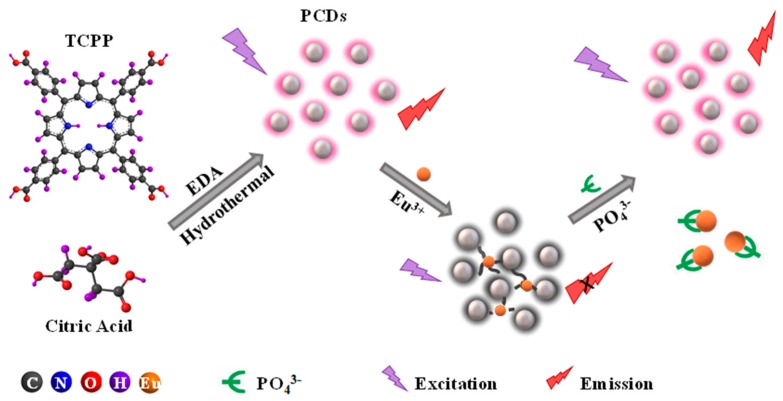
Schematic representation of the procedure for the preparation of porphin-based carbon dots (PCDs) and the sensing of phosphate.

Porphin and its derivatives are a class of organic molecule with the tetra-pyrrole core decorated with various functional groups on the peripheral, which have unique optical, electronic, and biochemical properties [42]. Porphin-based functional nanomaterials have been widely used in photothermal therapy [43,44], chemical sensing [45,46], light harvesting [47,48], and optoelectronic devices [49]. Meso-tetra (4-carboxyphenyl) porphin (TCPP, the molecular structure is shown in Scheme 1) is a type of macrocyclic organic molecule with large conjugated π-electron system [50]. The exploit of its unique structure allows the design and synthesis of red-emissive CDs via increasing the size of isolated sp^2^ domains, which makes TCPP an ideal precursor/carbon source. Moreover, four carboxyl groups on its peripheral benzene ring can increase the polarity of TCPP [51], giving rise to the excellent water solubility of CDs. Otherwise, considering that cancer cells have a slight acidic environment, the carboxyl groups would become electrostatically neutral, hence improving its uptake into lipophilic cell membranes [52,53]. The structure and properties of TCPP facilitate it as a potential candidate for preparation of CDs with long wavelength emission and application in biological fields.

Herein, by combining the excellent optical properties of CDs with the unique structure of TCPP, porphin-based carbon dots (PCDs) were designed and prepared by exploring TCPP as aromatic precursor, ethanediamine as nitrogen source, and citric acid as carbon source. On one hand, porphin induces the red photoluminescence of the prepared carbon dots, providing feasibility of low-background fluorescence imaging. On the other hand, the introduction of abundant carboxyl groups on their surface could act as reactive sites to coordinate with Eu^3+^. An off–on fluorescence-sensing system based on the novel PCDs as a fluorescent probe was established by exploiting the interaction between phosphate and Eu^3+^.

## 2. Experimental Section

### 2.1. Chemicals and Materials

Citric acid monohydrate, ethanediamine, and various kinds of compounds (Na_3_PO_4_·12H_2_O, KBr, NaF, NaClO, HCl, NaNO_3_, KI, CH_3_COONa·3H_2_O, CaCl_2_, KCl, NaCl, Na_2_SO_4_, Na_2_SO_3_, Na_2_CO_3_, NaHCO_3_, Cu(NO_3_)_2_·3H_2_O, Cd(NO_3_)_2_·4H_2_O, BaCl_2_·2H_2_O, MgCl_2_·6H_2_O, FeCl_3_·6H_2_O, FeCl_2_·4H_2_O, Zn(NO_3_)_2_·6H_2_O) were bought from Sinopharm Chemical Reagent Co., Ltd. (Shanghai, China). Na_2_S·9H_2_O was from Heng Xing Chemical Reagent Co. (Tianjin, China). Tris(hydroxymethyl)aminomethane and Eu(NO_3_)_3_·6H_2_O (99.99%) were from Aladdin Industrial Co. Ltd. (Shanghai, China). Meso-tetra (4-carboxyphenyl) porphin (97%) was purchased from Zhengzhou Alpha Chemical Co., Ltd. (Zhengzhou, China). DMEM high glucose medium and trypsin-EDTA were from Gibco Company (Grand Island, NY, USA). Fetal bovine serum was from Bovogen Biologicals Pty Ltd. (Melbourne, VIC, Australia). MTT (3-(4,5-dimethyl-2-thiazolyl)-2,5-diphenyl-2-H-tetrazolium bromide) was from MedChemExpress Company (Monmouth, NJ, USA). All other chemicals and reagents were of analytical grade and used without further purification. Tris-HCl buffer was disposed and adjusted in advance to control the acidity. The experimental water used in this study was secondary deionized water (18 MΩ·cm).

### 2.2. Apparatus

The morphology and size distribution of the products were performed by JEM-1200EX transmission electron microscopy (TEM) (JEOL, Tokyo, Japan). scanning and CSPM4000 atomic force microscope (AFM) (Benyuan Co. Ltd., Guangzhou, China). Fourier-transform infrared (FT-IR) spectra were performed on Nicolet iS50 instrument (Thermo Scientific, Madison, WI, USA) in the range of 500–4000 cm^−1^ wavenumbers. Kratos XSAM 800 X-ray photoelectron spectrometer (XPS) (Thermo Scientific, Madison, WI, USA) was used to collect XPS spectra. X-ray diffraction (XRD) is detected by D/MAX 2500/PC X-ray powder diffractometer (Rigaku Corporation, Tokyo, Japan) with Cu K_α_ radiation. Raman spectra were obtained with a laser excitation of 532 nm on DXR2 Raman Spectrometer (Thermo Scientific, Madison, WI, USA). UV-Vis absorption spectra and fluorescence spectra were performed on UV-2700 spectrophotometer (Shimadzu Corporation, Tokyo, Japan) and F-7000 fluorescence spectrophotometer (Hitachi High Technologies, Tokyo, Japan), respectively. FLS 1000 fluorescence spectrometer (Edinburgh Instruments, Livingston, UK) was used to measure fluorescence decay curves of CDs. HeLa cells were incubated in SCO6WE thermostatic incubator (SHEL LAB, Cornelius, OR, USA). Confocal fluorescence images of HeLa cells were performed on CTS SP8 confocal laser scanning microscopy (Leica, Wetzlar, Germany).

### 2.3. Synthesis of PCDs

Briefly, citric acid monohydrate (0.0480 g), meso-tetra (4-carboxyphenyl) porphin (0.0395 g), and ethylenediamine (300 μL) were mixed with 10 mL secondary deionized water. Then the prepared homogeneous solution was transferred into PTFE-lined autoclave for further hydrothermal treatment, which was set to 200 °C for 12 h. After that, the obtained mixture was cooled down to room temperature naturally and filtered through 0.22 μm filtration membrane to discard large particle residues. Afterwards, the PCDs were further depurated with dialysis against deionized water with cellulose ester membranes (Shanghai Yuanye Bio-Technology Co., Ltd., Shanghai, China, MWCO: 500–1000) for 3 days to remove the unreacted material. Carbon dots powder was obtained by rotary steaming and freeze-drying.

### 2.4. Cytotoxicity Assay and Cell Imaging

Firstly, HeLa cells were inoculated into a 96-well plate and cultured at 37 °C under 5% CO_2_ atmosphere for 24 h. Then, PCDs with different concentrations, which were set as 0, 50, 100, 150, 200, 250 μg mL^−1^, respectively, are added into each well and cultured for 12 h. Afterward, the medium is removed, 200 μL MTT (0.5 mg·mL^−1^) is added into each well, and incubated for a further 4 h. Then the medium was removed and 150 μL DMSO was added to each well. After gently shaking for 10 min, the absorbance at 570 nm of each well is measured using a microplate reader (Bio-RADiMark^TM^, Hercules, CA, USA).

The cellular uptake experiment was carried out as follows. HeLa cells were incubated with DMEM high glucose medium containing 10% of fetal bovine serum, 100 units mL^−1^ penicillin, and 100 mg mL^−1^ streptomycin at 37 °C with 5% CO_2_ for 24 h. Then PCDs (50 μg·mL^−1^ in PBS) were added into the medium. After incubation for 12 h, the cells were rinsed twice in PBS buffer, the confocal fluorescence images of HeLa cells were taken on a confocal laser scanning microscopy with the laser excitation wavelength at 552 nm.

### 2.5. Formation of PCDs-Europium Aggregates (PCDs-Eu^3+^)

A total of 1 mg of PCDs powder was dissolved in 50 mL of Tris–HCl buffer solution (0.05 mol L^−1^, pH 7.8). A total of 0.02 mg mL^−1^ of PCDs solution (Tris–HCl buffer, pH 7.8) was allowed to react with different concentrations of Eu^3+^ solution (0, 5, 10, 15, 20, 25, 30, 35, 40 μmol·L^−1^) (Tris–HCl buffer, pH 7.8) at room temperature for 15 min. The fluorescence of this system was recorded at λ_ex_/λ_em_ = 375/645 nm. By comparison, 30 μmol·L^−1^ Eu^3+^ was selected for further use.

### 2.6. The Sensing of Phosphate (PO_4_^3−^)

A total of 1.0 mmol L^−1^ of phosphate stock solution was prepared in Tris–HCl buffer. Different concentrations of phosphate solutions (i.e., 0, 0.02, 0.04, 0.06, 0.08, 0.10, 0.20, 0.40, 0.60, 0.80, 1.00 μmol·L^−1^) were added into the PCDs-Eu^3+^ aggregates. The fluorescence intensity at λ_ex_/λ_em_ = 375/645 nm was measured after 15 min of mixture. The preparation of PCDs and phosphate sensing process were illustrated as Scheme 1.

### 2.7. Sample and Sample Pretreatment

Artificial lake water (from Qingdao University) was used as sample and insoluble particles were filtered by a 0.22 μm pore size membrane before using. Human whole blood samples (provided by healthy volunteers from the Hospital of Qingdao University) were firstly injected into the Tris–HCl buffer; after intense stirring for 5 min, the suspension was centrifuged at 5000 rpm for 10 min to remove all cell debris, and the supernatant fluid was collected for further determination. Urine and saliva samples (provided by healthy volunteers from the Hospital of Qingdao University) were diluted with Tris–HCl buffer and then filtered through a 0.22 μm pore size filter membrane for further analysis. Finally, spiking recovery experiments were carried out by adding a certain amount of phosphate standard solutions into the resultant real samples.

## 3. Results and Discussion

### 3.1. Characterization of PCDs

**Figure 1:**
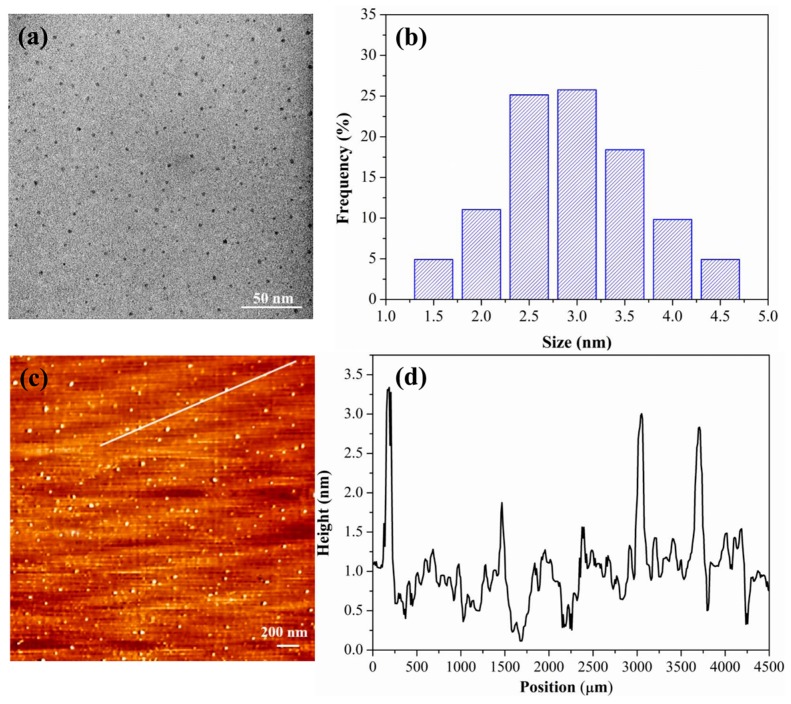
Transmission electron microscopy (TEM) image (**a**) and the size distribution (**b**) of PCDs; Atomic force microscope (AFM) image on mica substrates (**c**) with the height profile along the line in the image (**d**) of PCDs.

The morphological structures of PCDs were characterized by TEM and AFM. As shown in Figure 1a, the PCDs were almost spherical, well dispersed, and displayed a 1.5–4.5 nm narrow particle size distribution with a calculated average diameter of ca. 3.0 nm. AFM images demonstrated that the height of PCDs was in a range of 1.0–3.5 nm, which was consistent with the TEM results. The XRD curve in the Supplementary Materials Figure S1a showed a typical broad peak at 2θ = 21.42°, revealing that PCDs were amorphous in structure. From Figure S1b, two typical peaks at 1359 cm^−1^ (D band) and 1595 cm^−1^ (G band) were observed in the Raman spectrum, representing the disordered structures or defects (sp^3^ carbon) of the PCDs and crystalline graphitic sp^2^ carbon, respectively. The intensity ratio of D band to G band (I_D_/I_G_) was derived to be 0.67, showing an amorphous carbon phase of PCDs.

**Figure 2:**
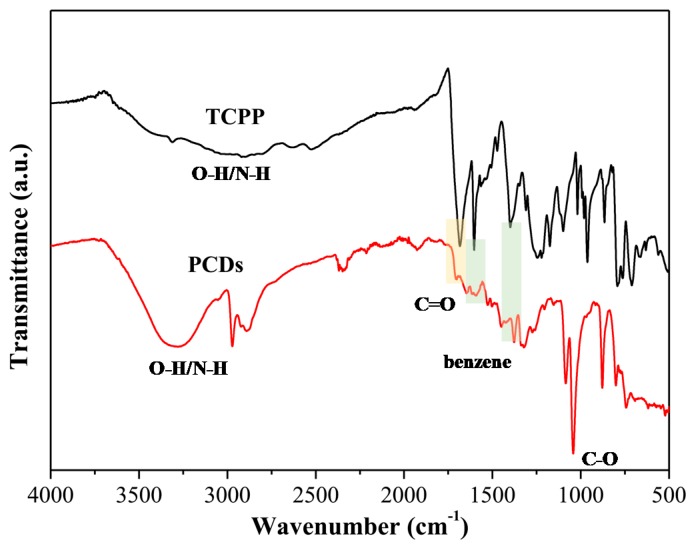
FT-IR spectra of meso-tetra (4-carboxyphenyl) porphin (TCPP) and PCDs.

FT-IR and XPS spectra were introduced to investigate the composition and surface chemistry of PCDs. Figure 2 shows the FT-IR spectrum of TCPP and PCDs. In TCPP, the broad peak at 2910 cm^−1^ was ascribed to O–H/N–H groups. The stretching vibrations located at 3457 and 1684 cm^−1^ were due to O–H and C=O groups of the carboxyl acid group, 1600 and 1392 cm^−1^ were the characteristic absorption of the benzene ring. For PCDs, the strong stretching vibration peak of O–H/N–H groups (3290 cm^−1^), and the stretching vibrations of C=O (1644 cm^−1^) and C–O (1085 cm^−1^) indicated that the existence of hydroxyl, amino, and carboxyl groups around the surface of PCDs. These abundant hydrophilic groups ensure PCDs exhibit good solubility in aqueous solution. Besides, the stretching vibrations of C=C at 1620 cm^−1^, C–C at 1482 cm^−1^, C=N at 1592 cm^−1^, and C–N at 1372 cm^−1^ demonstrated the conjugated aromatic structure and the oxygen-rich and nitrogen-rich functional groups were involved in PCDs; TCPP as a precursor had successfully formed into PCDs with its conjugated structure and functional groups.

**Figure 3:**
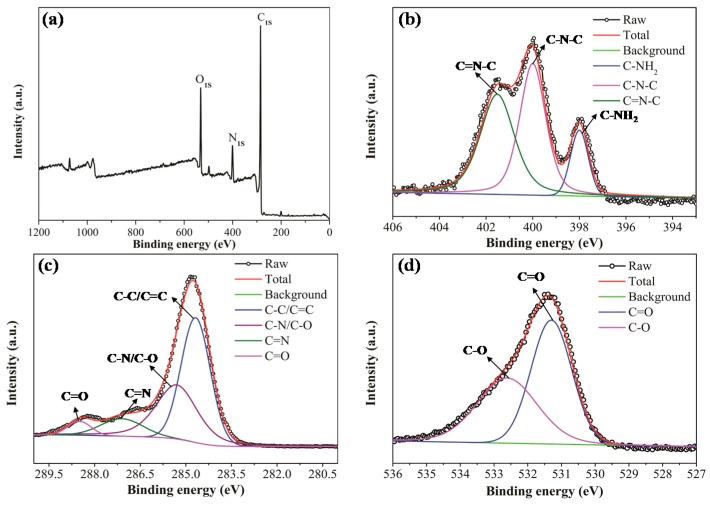
(**a**) XPS spectra and (**b**) N_1s_, (**c**) C_1s_, and (**d**) O_1s_ peaks of the as-prepared PCDs.

The XPS measurement (Figure 3) reveals three prominent features of C_1s_ peak at 285 eV, O_1s_ peak at 531 eV, and N_1s_ peak at 400 eV, with the atomic percentages of 71.15%, 13.99%, and 14.86%, respectively. The N_1s_ spectrum in Figure 3b could be peak-differentiated to three types of nitrogen at 398.0, 400.0, and 401.5 eV, respectively, associated with C=N–C, C–N–C, and C–NH_2_, indicating that pyridine nitrogen, pyrrolic nitrogen, and alkyl ammonium were involved in PCDs. The typical C_1s_ spectrum (Figure 3c) shows four distinct peaks at 284.7, 285.3, 287.1, and 288.5 eV, which are ascribed to C–C/C=C, C–N/C–O, C=N, and C=O, respectively, indicating the existence of conjugated structure. The two resolved peaks at 531.1 and 532.6 eV in the O_1s_ spectrum (Figure 3d) are attributed to C=O and C–O bands, respectively. The XPS data agreed well with the FT-IR results, further revealing that nitrogen-doping, functional groups, and conjugated structure were all involved in PCDs.

### 3.2. Optical Properties of PCDs

**Figure 4:**
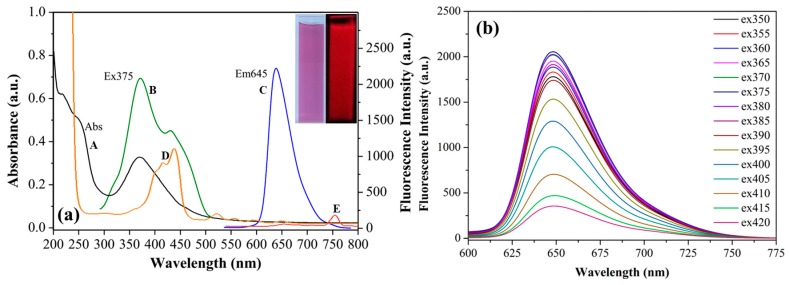
(**a**) UV-Vis absorption (A), fluorescence excitation (λ_ex_, B) and emission (λ_em_, C) spectra of PCDs in aqueous solution; UV-Vis absorption (D) and fluorescence emission (λ_em_, E) spectra of TCPP in aqueous solution; The inset: The photographs of PCDs aqueous solution under daylight (left) and UV light (right). (**b**) Fluorescence emission spectra of PCDs with excitation wavelengths from 350 to 420 nm in 5 nm intervals.

The spectrum of UV-Vis absorption is shown in Figure 4a. The absorption in 200–300 nm was attributed to the π-π* transition of the aromatic ring sp^2^ domain. While the absorption band centered at 375 nm was attributed to n-π* transition of C=O, related to the surface adsorption of PCDs [54]. The fluorescence emission spectra at various excitation wavelengths are described in Figure 4b. Interestingly, PCDs exhibited an unchanged emission peak at 645 nm, although the intensity was varied when the excitation wavelength changed from 350 to 420 nm (with an interval of 5 nm), suggesting excitation-independence emission. When PCDs were excited at the wavelength of 375 nm, the maximum fluorescence emission intensity at 645 nm was obtained. In comparison, the UV-Vis absorption and PL spectrum of TCPP were also measured, whereby typical Soret bands of porphin absorption are observed. Meanwhile, TCPP showed slight fluorescence emission compared with PCDs. The changes of UV-Vis absorption and PL spectrum indicated that TCPP had transformed into PCDs in the hydrothermal reaction. The quantum yield of PCDs was measured to be 2% by using Rhodamine B as a reference (31%, in water).

To explain the possible photoluminescence mechanism of red-emissive CDs, several explanations were concluded as follows. In the visible region, the fluorescence excitation spectrum of PCDs (λ_em_: 645 nm) were well overlapped with the UV-Vis absorption spectrum, illustrating that the red shift of emission was resulted from the surface structures and states [55]. Consequently, the photoluminescence mechanism can be attributed to the surface molecular state-related fluorescence [56]. It was speculated that the excitation-independent fluorescence emission phenomenon could be attributed to the relative narrow size distribution, the uniform emissive traps at the surface [57], and the direct recombination of excitons of PCDs because of N atom doping [58]. The abundant hydroxyl, carboxyl, and amino groups on the surface of PCDs tended to form a conjugated π domains structure, and finally resulted in the surface state-related emissive trap states, reducing the energy band gap of π-π* transition, changing the electronic structure of PCDs [59,60]. So the relatively uniform surface state emissive traps give rise to the excitation independent emission behavior [61]. Thus, the photoluminescence of PCDs was believed to be dominated by the surface molecular state emission mechanism.

As a conclusion, employing polyaromatic chemicals as precursors or the enlargement of the sp^2^ domain size of CDs is an active approach to red-shift the emissive photoluminescence [15]. TCPP with conjugated aromatic structure would, consequently, lower the energy gap by increasing the size of sp^2^ domains [16]. It is well known that heteroatomic doping is confirmed to be capable of regulating the photoluminescent property of CDs. Herein, as an electron donor, nitrogen atom could bond with carbon atom, thus disordering the structure of carbon rings and creating a new energy gap between the excited state and the ground state through the radiative recombination. Therefore, nitrogen doping can narrow the bandgap, generating the emission of strong red fluorescence [62,63].

The photoluminescent decay profiles of PCDs are displayed in Figure S2. The decay curves of PCDs were well fitted to a single-exponential model for luminescence decay with fluorescence lifetimes of 8.93 ns. On one hand, this value is larger than those in literature [4,64], thus the as-prepared PCDs have potential applications in lifetime-based bioimaging and biosensing. On the other hand, as reported, the single-exponential model for luminescence decay of CDs is in accord with the excitation-independent emission property, implying relatively uniform fluorescence radiative processes [58,65].

The aqueous solution of prepared PCDs was lavender, transparent under day light, and it exhibited strong red luminescence on exposure of UV light at 365 nm, as shown in the inset of Figure 4a. As illustrated in Figure S3, fluorescence intensity of PCDs remained almost constant even after continuous excitation for 8000 s and storage for 90 days, which demonstrated the outstanding stability and anti-photo-blenching of PCDs. This ensured PCDs as a fluorescent probe that was feasible and stable in aqueous solutions.

### 3.3. Cytotoxicity Assay and Cell Imaging

Herein, HeLa cells were chosen as a model. Standard MTT assay was carried out to test the cytotoxicity and biocompatibility of PCDs. From Figure S4a, over 80% cell viability was obtained after 12 h incubation of HeLa cells with PCDs in concentrations under 100 μg·mL^−1^. This confirmed the low cytotoxicity and excellent biocompatibility of the prepared PCDs, suggesting that it could be safely used for in vitro cell imaging.

**Figure 5:**
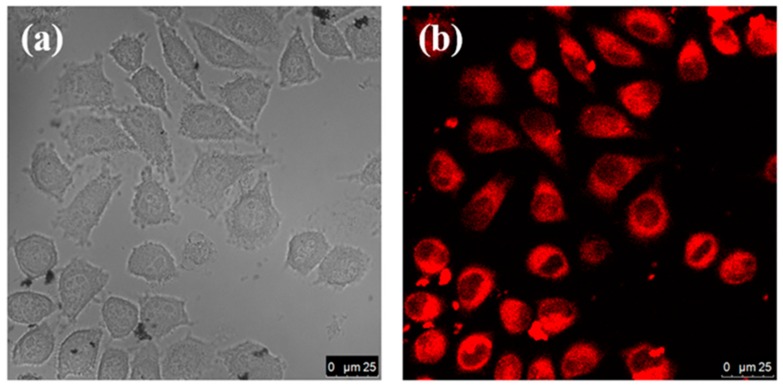
Confocal fluorescence microscopy images of HeLa cells incubated with PCDs under bright field (**a**) and the excitation wavelength of 552 nm (**b**).

To evaluate the cell imaging ability of PCDs, the cellular uptake experiment was performed. After incubation with PCDs (50 μg·mL^−1^) for 12 h, confocal fluorescence microscopy images (Figure 5) of HeLa cells were taken under excitation at 552 nm and bright red emission from the intracellular region was observed, which could be obviously distinguished from the control cells (Figure S4c). In this work, the high quality and high resolution red-emissive images were obtained, verifying that the PCDs can penetrate cells and efficiently avoid the autofluorescence interference of the biological matrix and the damage to biological samples; indicating that PCDs can be used as a direct and efficient cell labelling probe. These above results showed that PCDs had great potential in biological applications.

### 3.4. Fluorescence Quenching Behavior of PCDs to Eu^3+^

**Figure 6:**
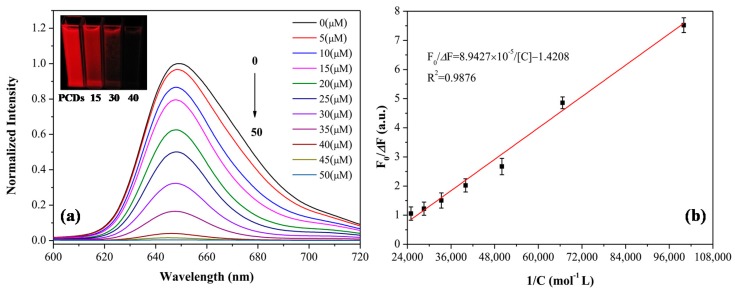
(**a**) Fluorescence of the PCDs quenched by different concentrations of Eu^3+^ (0, 5, 10, 15, 20, 25, 30, 35, 40, 45, 50 µmol·L^−1^); inset: The photographs of PCDs (left) and PCDs in the presence of 15, 30, 40 µmol·L^−1^ Eu^3+^ (from left to right) on the exposure of UV light. (**b**) Modified Stern–Volmer representation of PCDs after titration with Eu^3+^.

As one kind of important rare earth element, Eu^3+^ shows a certain affinity with the oxygen atom donors, which can coordinate with carboxylate groups on the surface of PCDs [10,66]. Here, when Eu^3+^ was titrated into the PCDs solution, a sharp decrease of fluorescence intensity could be seen. As shown in Figure 6, adding increasing concentrations of Eu^3+^ (0, 5, 10, 15, 20, 25, 30, 35, 40, 45, 50 µmol L^−1^) into the PCDs solution resulted in gradually decreasing fluorescence of PCDs at λ_ex_/λ_em_ = 375/645 nm, and the fluorescence intensity reached a plateau at a Eu^3+^ concentration > 40 µmol·L^−1^. At the same time, an obvious red color fading could be seen from Figure 6a inset on the exposure of UV lamp (365 nm). The fluorescence change would be observed by visual fluorescence color change. Nearly 100% of the fluorescence was quenched by 40 µmol·L^−1^ Eu^3+^, which resulted in the turn-off of fluorescence through the aggregation. A total of 40 µmol·L^−1^ Eu^3+^ was chosen for the following experiments. As seen from Figure S5, the aggregation were observed clearly, which was much larger than the size of PCDs, verifying that PCDs-Eu^3+^ aggregates were formed. Consequently, the fluorescence of PCDs turned off.

To gain a deep look into the photoluminescent quenching mechanism, the time-resolved photoluminescent spectra of PCDs-Eu^3+^ aggregates was also measured. The fluorescence lifetimes of PCDs and PCDs-Eu^3+^ were displayed in Figure S2, which showed the lifetime of 8.93 and 9.07 ns, respectively. In theory, in the static quenching process, whether in the absence (τ_0_) or presence (τ) of Eu^3+^ as quencher, the fluorescence lifetimes equal to a constant τ value (τ_0_/τ = 1). Herein the same fluorescent lifetime further confirmed that the quenching of PCDs by Eu^3+^ acted up to the static quenching mechanism. According to the report, the quenching can be depicted by the Stern–Volmer equation (*F_0_*/*F* − 1 = K_sv_·[*C*]) or a modified Stern–Volmer equation (*F_0_*/*∆F* = 1/(f_a_K_sv_·[*C*] + 1/f_a_), where *∆F* = (*F_0_* − *F*), F_0_, and F are the fluorescence intensity of PCDs in the absence and presence of Eu^3+^; [C] represents the concentration of Eu^3+^; K_sv_ is the Stern–Volmer quenching constant; f_a_ is the fraction of original fluorescence that interacts with the quencher [9,67]. As shown in Figure S6, the quenching behavior didn’t fit well with the typical Stern–Volmer curve. This upward curvature suggested that the fluorophores present in the PCDs were not all equally coordinated with the Eu^3+^ and only parts of them were affected by Eu^3+^ and exhibited quenching of the fluorescence. In order to consult whether this effect results in the curvature of the typical Stern–Volmer plot, a modified Stern–Volmer model was used (Figure 6b). In the range of 10–40 μmol·L^−1^, a linear relationship was observed, which fitted well with the modified Stern–Volmer equations (*F_0_*/*∆F* = 8.9427 × 10^−5^/[*C*] − 1.4208, R^2^ = 0.9876). The K_sv_ value was derived to be ~15,900 mol^−1^·L, corresponding to k_q_ value of 1.8 × 10^12^ mol·L^−1^·s^−1^, which is considerably bigger than the maximum of k_q_ for the diffusion-controlled quenching process (ca. 10^10^·mol·L^−1·^s^−1^), verifying that the dominative quenching mechanism is due to the static quenching [68]. The above results indicated that the quenching was suggested to be static quenching and an aggregate was formed between PCDs and Eu^3+^.

### 3.5. Mechanism of Fluorescent Response to Phosphate

Phosphate plays pivotal roles in living organisms and the environment. On one hand, phosphate is a vital constituent of biosystems, which is an essential constituent of nucleic acid. The abnormal concentrations of phosphate in biological fluids (e.g., blood, saliva, urine) will cause physiological dysfunction, such as hyperparathyroidism and cardiovascular complications [69,70]. On the other hand, ecosystems are sensitive to the levels of available phosphate; as an indicator of organic pollution [71], excessive concentration of phosphates in natural water will result in eutrophication. Therefore, the quantitative determination of phosphate is of vital importance.

As mentioned above, when phosphate is introduced, Eu^3+^ has a stronger affinity for phosphate than carboxylate groups [62,72]. The addition of phosphate to PCDs-Eu^3+^ aggregates can recover the fluorescence intensity with a five-fold enhancement of this system, due to the desorption of Eu^3+^ from the surface of the PCDs, by utilizing the competition between the oxygen atom donors from the phosphate groups and those from the carboxylate groups on the surface of PCDs for Eu^3+^. PCDs redispersed in the aqueous solution, as seen from Figure S5b, further confirmed the above conclusion.

In theory, Eu^3+^ exhibits a considerable affinity to the oxygen atom donors; the fluorescence response of other oxygen-containing groups including CH_3_COO^−^, HCO_3_^−^, ClO^−^, SO_3_^2−^, CO_3_^2−^, SO_4_^2−^, and NO_3_^−^ were also investigated under the same experimental conditions. Otherwise, the fluorescence response of PCDs-Eu^3+^ towards other cations (Mg^2+^, Ba^2+^, Na^+^, Ca^2+^, K^+^) and anions (F^−^, S^2−^, Br^−^, Cl^−^, I^−^) were also studied (Figure S7). As expected, no apparent fluorescence variation occured with the addition of these groups, indicating that other ions cannot recover the fluorescence of PCDs-Eu^3+^. Only phosphate could selectively bind with the PCDs-Eu^3+^ system.

As shown in Figure S8, the fluorescence spectra of PCDs-Eu^3+^, by titrating with different concentrations of phosphate, were recorded under the optimized experimental conditions. The fluorescence intensity of PCDs-Eu^3+^ was gradually increased with the increasing concentration. As the concentration increased up to 60.0 μmol·L^−1^, a total 100% fluorescence restore could be obtained. Moreover, to some extent, this fluorescence recovery process could be visually observed by naked eyes under the illumination of UV light, as shown in the Figure S8 inset.

**Figure 7:**
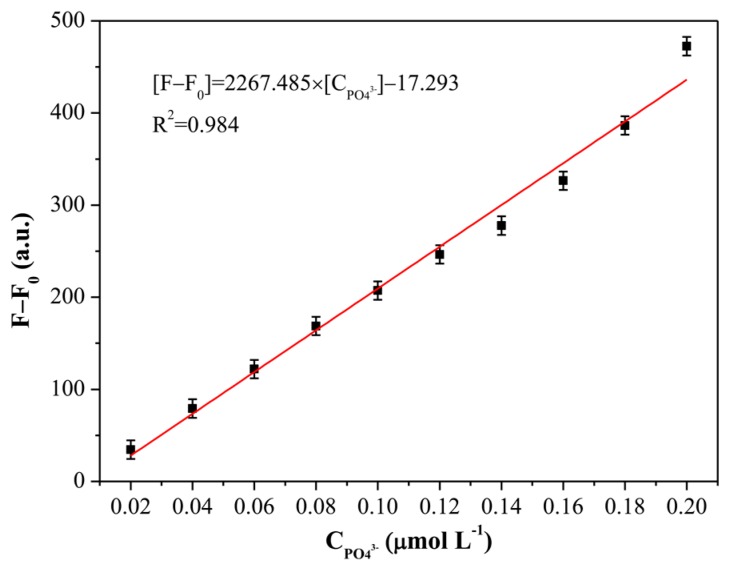
The linear relationship of F-F_0_ versus the concentration of phosphate (0.02–0.20 μmol·L^−1^). PCDs, 0.02 mg·mL^−1^; Eu^3+^, 30.0 μmol·L^−1^; Tris–HCl buffer, pH 7.8.

In the range from 0.02 to 0.20 μmol·L^−1^, a linear relationship with a correlation coefficient of 0.984, by plotting F-F_0_ (F, F_0_ are the fluorescence intensities of the PCDs-Eu^3+^ at λ_ex_/λ_em_ = 375/645 nm in the absence and presence of phosphate) versus the concentration of phosphate (C, μmol·L^−1^), was obtained as: F-F_0_ = 2267.485[C] − 17.293 (Figure 7). The detection limit of 3.59 × 10^−3^ μmol L^−1^ was evaluated with a signal-to-noise ratio of 3. The relative standard deviation (RSD) was 1.42% (phosphate concentration: 0.1 μmol·L^−1^, *n* = 11). As illustrated in Table 1, compared with currently reported fluorescence methods in the literature [10,73,74,75,76,77], the linear range and detection limit were superior to other reports.

### 3.6. Selectivity Study for Phosphate Detection

The selectivity of this sensing platform was carried out before application in real sample analysis; the control experiments about the fluorescence responses towards potential interferences from various coexisting ions were investigated. The results were illustrated in Figure S9; no obvious interference was seen on the fluorescence of the PCDs-Eu^3+^-PO_4_^3−^ system in the presence of a 100-fold excess of Br^−^, SO_3_^2−^, S^2−^, ClO^−^, NO_3_^−^, HCO_3_^−^, Cl^−^, AC^−^, SO_4_^2−^, K^+^, Na^+^, CO_3_^2−^, and I^−^, and a 60-fold excess of F^−^, Zn^2+^, Mg^2+^, Fe^2+^, Fe^3+^, Ba^2+^, Ca^2+^, Cu^2+^, and Cd^2+^, confirming that this system was effective for the selective detection of phosphate, with good anti-interference ability to coexisting ions.

To demonstrate the practical usefulness of the proposed method, real samples including artificial lake, saliva, urine, and blood serum were completed, and the results are listed in Table 2. In practice, the concentration of phosphate ion human serum is in the range of 0.81–1.45 mmol·L^−1^ [78]. The blood serum sample determined was 0.98 ± 0.30 mmol·L^−1^, which was at the normal level. The phosphate in saliva estimated by this method was 4.02 ± 0.66 mmol·L^−1^, which fitted well with the literature report of 3.22–5.90 mmol·L^−1^ [79]. Compared with the traditional biological fluid (e.g., serum), saliva determination has the advantages of being noninvasive, cheap, and patient friendly, offering a promising diagnostics technique. To further evaluate the feasibility of this sensing system, spiking recoveries were performed for the real samples. As illustrated in Table 2, different concentrations of phosphate were spiked into these samples; the results obtained were satisfactory over the range of 96.36–102.85%, further proving the practical feasibility of this sensing platform.

## 4. Conclusions

In conclusion, porphin-based carbon dots were prepared by using meso-tetra (4-carboxyphenyl) porphin with a conjugated aromatic structure as carbon precursor, which offers a promising candidate for the development of a fluorescence labelling probe in biological imaging. By europium ion regulation, a simple phosphate detection “turn off–on” method was established, with PCDs as the fluorescence sensing probe. It was applied for the quantitative detection of phosphate with high sensitivity, high selectivity, and excellent stability in biological fluids, such as human whole blood, serum, urine, saliva samples, and environmental water samples. This work provided not only a novel strategy for fabrication of CDs with red emission, but also a promising platform for diagnostic and environmental monitoring, and it also showed great potential in biomedical fields.

## Figures and Tables

**Table 1:** A comparison of different systems for phosphate determination.

Method	Linear Range/μmol L^−1^	LOD/μmol·L^−1^	Ref.
Amino derivative of UiO-66	5–150	1.25	73
Europium-based metal-organic framework	3–30	6.62	74
Single-layered graphene quantum dots	0.2–30	0.1	75
Metal- organic frameworks-based nanocomposite	80–400	2	76
Graphene quantum dots combined with europium ions	0.5–190	0.1	77
Europium-adjusted carbon dots	0.4–15	5.1 × 10^−2^	10
PCDs	0.02–0.2	3.59 × 10^−3^	This work

**Table 2:** The determination results of phosphate for four kinds of sample matrixes (*n* = 3, 95% confidence level).

Practical Samples	Original (mmol·L^−1^)	Spiked (mmol·L^−1^)	Found (mmol·L^−1^)	Recovery (%)
Artificial lake	(0.51 ± 0.02) × 10^−3^	0.25 × 10^−3^	(0.75 ± 0.02) × 10^−3^	96.47 ± 5.82
		0.40 × 10^−3^	(0.90 ± 0.01) × 10^−3^	98.86 ± 2.75
		0.60 × 10^−3^	(1.09 ± 0.04) × 10^−3^	96.36 ± 6.20
Saliva	4.02 ± 0.66	2.00	6.01 ± 0.05	100.12 ± 2.75
		4.00	7.95 ± 0.07	98.60 ± 1.66
		6.00	10.16 ± 0.25	102.46 ± 3.90
Urine	26.40 ± 2.86	12.50	39.17 ± 0.58	101.94 ± 4.78
		30.00	56.65 ± 1.61	100.85 ± 3.08
		40.00	65.35 ± 0.66	97.37 ± 1.61
Blood serum	0.98 ± 0.30	0.50	1.49 ± 0.10	102.85 ± 2.26
		1.50	2.50 ± 0.05	101.72 ± 3.22
		2.00	2.94 ± 0.04	98.18 ± 2.03

## Supplementary Materials

The following are available online at https://www.mdpi.com/2079-4991/10/2/326/s1, Figure S1: The XRD (**a**) and Raman spectra (**b**) of PCDs, Figure S2: The photoluminescent decay profiles and lifetimes of PCDs and PCDs-Eu^3+^, Figure S3: The variation of fluorescence intensity for PCDs with irradiation time (λ_ex_/λ_em_ = 375/645 nm), Figure S4: Cell viability of HeLa cells at different concentrations of PCDs (**a**); Confocal fluorescence microscopy images of HeLa cells without labeling under bright field (**b**) and the excitation wavelength of 552 nm (**c**), Figure S5: TEM images of PCDs-Eu^3+^ (**a**) and PCDs-Eu^3+^-PO_4_^3−^ (**b**), Figure S6: The relationship between F_0_/F-1 and the concentration of Eu^3+^, Figure S7: Fluorescence variations of PCDs-Eu^3+^ to different kinds of anions and cations, Figure S8: The emission spectra of PCDs-Eu^3+^ upon the addition of various amounts of phosphate (0.1–60.0 μmol·L^−1^); Inset: the photographs of PCDs-Eu^3+^ in the presence of 0, 4, 10, 20, 40, 60 µmol·L^−1^ phosphate and the original PCDs(from left to right) under the illumination of daylight and UV light, Figure S9: Fluorescence response of PCDs-Eu^3+^-PO_4_^3−^ under the co-existing of other ions (PO_4_^3−^, 50 µmol L^−1^; Br^−^, SO_3_^2−^, S^2−^, ClO^−^, NO_3_^−^, HCO_3_^−^, Cl^−^, AC^−^, SO_4_^2−^, K^+^, Na^+^, CO_3_^2−^, I^−^, 5000 µmol L^−1^; F^−^, Zn^2+^, Mg^2+^, Fe^2+^, Fe^3+^, Ba^2+^, Ca^2+^, Cu^2+^, Cd^2+^, 300 µmol L^−1^.).
