# Supplementary material for: Porphin-Based Carbon Dots for “Turn Off–On” Phosphate Sensing and Cell Imaging
Source: Nanomaterials (Basel). 2020 Feb 14;10(2):326. doi: 10.3390/nano10020326 (PMC7075202; doi:10.3390/nano10020326)
Supplement: Supplementary file 1 [file nanomaterials-10-00326-s001.pdf]

Supporting Information

Porphin-based Carbon Dots for "Turn Off–On" Phosphate Sensing and Cell Imaging

Jing Wu †, Wenjing Wang † and Zonghua Wang *

College of Chemistry and Chemical Engineering, Shandong Sino-Japanese Center for Collaborative Research of Carbon Nanomaterials, Qingdao University, Qingdao 266071, China
* Correspondence: wangzonghua@qdu.edu.cn; Tel.: +86-532-85950873
† These authors contributed equally to this work.

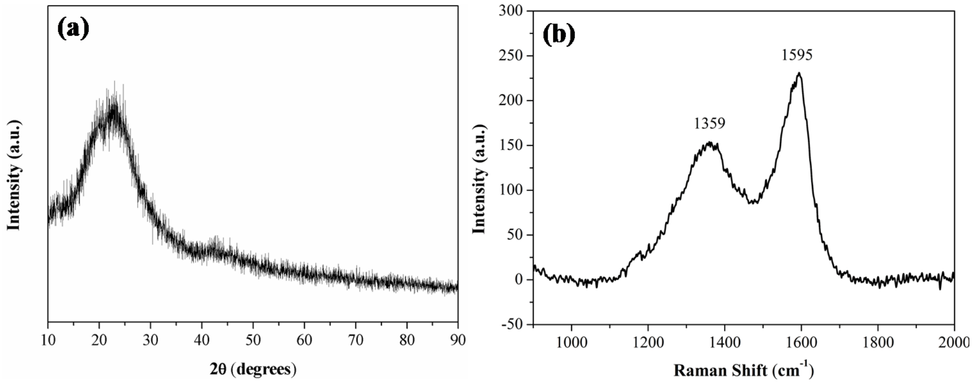

Figure S1. The XRD (**a**) and Raman spectra (**b**) of PCDs.

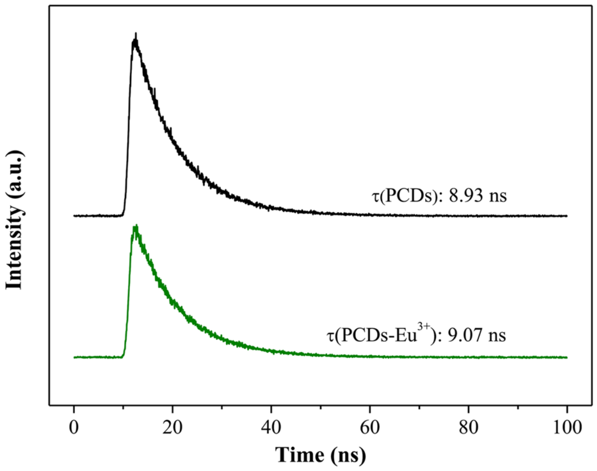

Figure S2. The photoluminescent decay profiles and lifetimes of PCDs and PCDs-Eu^{3+}.

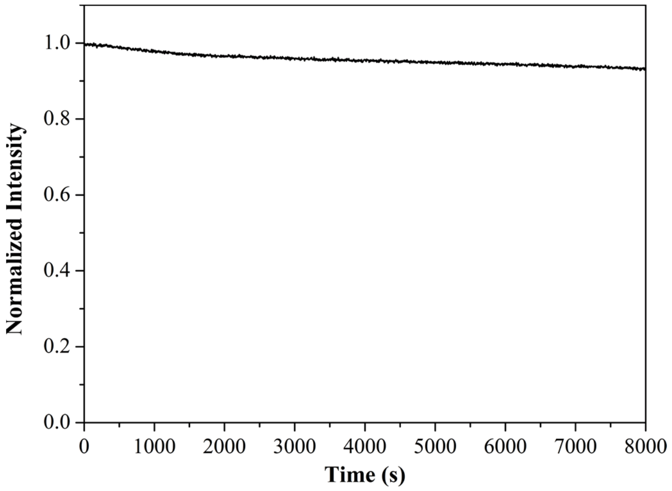

Figure S3. The variation of fluorescence intensity for PCDs with irradiation time ($\lambda_{ex}/\lambda_{em}$ = 375/645 nm).

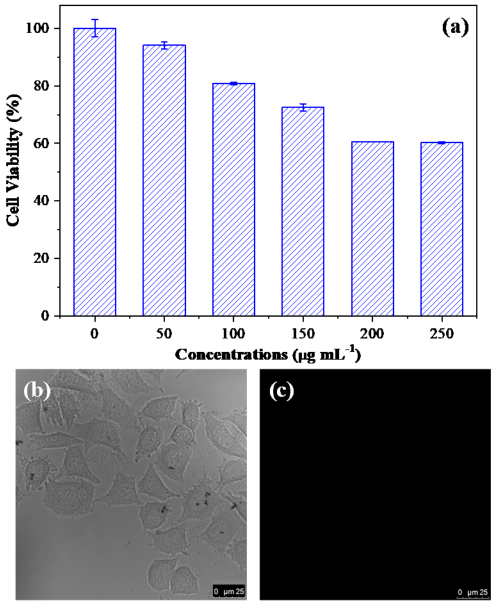

Figure S4. Cell viability of HeLa cells at different concentrations of PCDs (0, 50, 100, 150, 200, 250 μg mL^{-1}) (**a**); Confocal fluorescence microscopy images of HeLa cells without labeling under bright field (**b**) and the excitation wavelength of 552 nm (**c**).

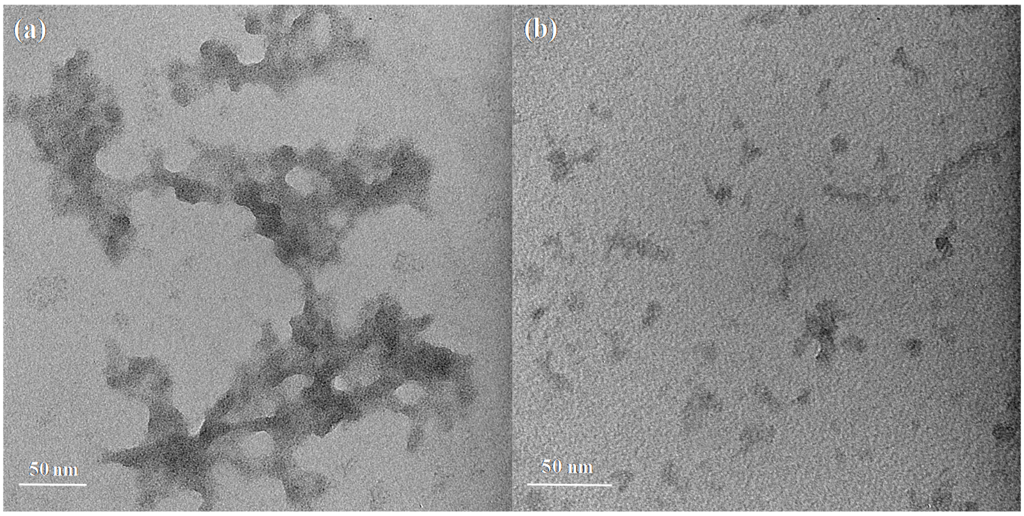

Figure S5. TEM images of PCDs-Eu^{3+} (**a**) and PCDs-Eu^{3+}-PO_4^{3-} (**b**).

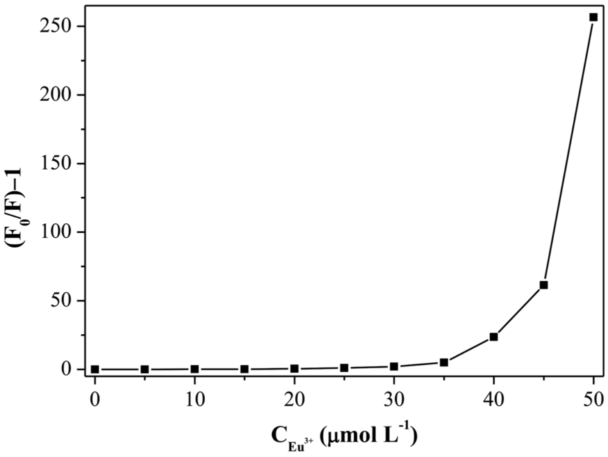

Figure S6. The relationship between $F_0/F - 1$ and the concentration of Eu^{3+}.

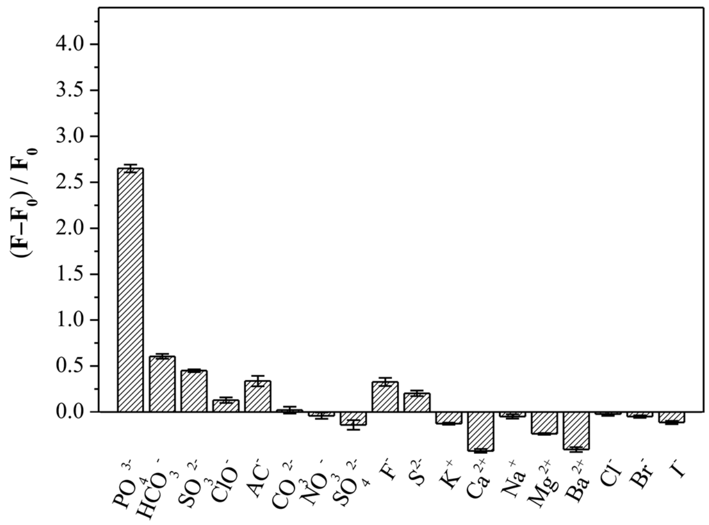

Figure S7. Fluorescence variations of PCDs-Eu^{3+} to different kinds of anions and cations. Concentrations: PO_4^{3-}: 0.05 mmol L^{-1}; F^-: 0.5 mmol L^{-1}; HCO_3^-, SO_3^{2-}, ClO^-, CH_3COO^- (AC^-), CO_3^{2-}, NO_3^-, SO_4^{2-}, S^{2-}, K^+ Ca^{2+}, Na^+, Mg^{2+}, Ba^{2+}, Cl^-, Br^-, I^-: 5.0 mmol L^{-1}. All data are collected at λ_{ex} 375.0 nm. PCDs, 0.02 mg mL^{-1}; Eu^{3+}, 30.0 μmol L^{-1}; Tris–HCl buffer, pH 7.8.

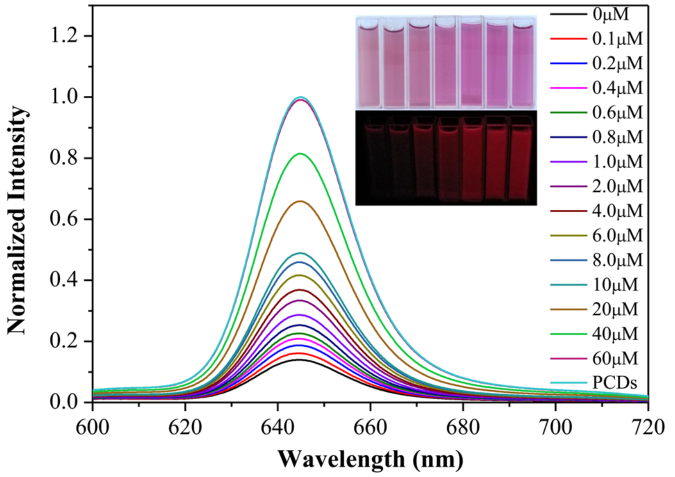

Figure S8. The emission spectra of PCDs-Eu^{3+} upon the addition of various amounts of phosphate (0.1–60.0 μmol L^{-1}); Inset: the photographs of PCDs-Eu^{3+} in the presence of 0, 4, 10, 20, 40, 60 μmol L^{-1} phosphate and the original PCDs (from left to right) under the illumination of daylight and UV light.

nanomaterials

MDPI

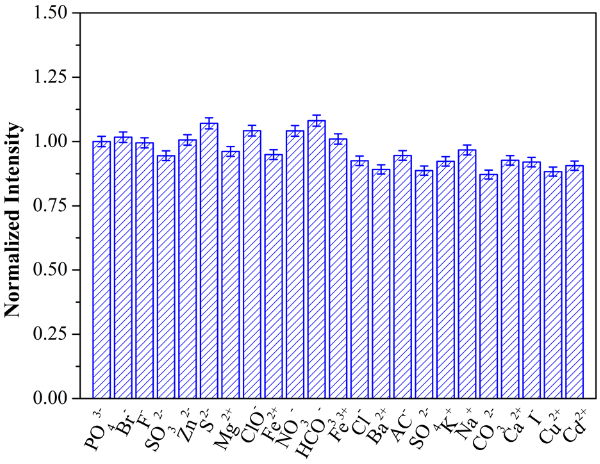

Figure S9. Fluorescence response of PCDs-Eu^{3+}-PO_4^{3-} under the co-existing of other ions (PO_4^{3-}, 50 μmol L^{-1}; Br^-, SO_3^{2-}, S^{2-}, ClO^-, NO_3^-, HCO_3^-, Cl^-, AC^-, SO_4^{2-}, K^+, Na^+, CO_3^{2-}, I^-, 5000 μmol L^{-1}; F^-, Zn^{2+}, Mg^{2+}, Fe^{2+}, Fe^{3+}, Ba^{2+}, Ca^{2+}, Cu^{2+}, Cd^{2+}, 300 μmol L^{-1}.) All data are collected at $\lambda_{ex}/\lambda_{em}$ = 375/645 nm. PCDs, 0.02 mg mL^{-1}; Eu^{3+}, 30.0 μmol L^{-1}; Tris–HCl buffer, pH 7.8.
